# Differences in medical students’ academic interest and performance across career choice motivations

**DOI:** 10.5116/ijme.56a7.5124

**Published:** 2016-02-15

**Authors:** Kyong-Jee Kim, Jee Y. Hwang, Bum S. Kwon

**Affiliations:** 1 Department of Medical Education, School of Medicine, Dongguk University, Korea; 2 Department of Rehabilitation Medicine, School of Medicine, Dongguk University, Korea

**Keywords:** Academic interest, medical students, performance, career choice, motivations

## Abstract

**Objectives:**

To investigate medical students’ career choice motivation and its relationship with their academic interest and performance.

**Methods:**

We conducted a cross-sectional study in a sample (n=207) of medical students at a private medical school in Korea, stratified by year of medical course. Data about participant demographics, career choice motivation and academic interest were collected using a self-report questionnaire. The item on career choice motivation enquired about the respondents’ main reason for applying for medical school among 8 possible response options, which comprised two components of career choice motivation: intrinsic and extrinsic. The participants’ levels of academic interest were measured in a Likert-type question. Participants’ academic interest and Grade Point Averages (GPAs) were compared across the groups of different career motivations along with analyses of their admission scores for baseline comparisons.

**Results:**

A total of 195 students completed the questionnaire (94%response rate). Seventy-four percent, (n=145; the intrinsic group) of the participants chose reasons related to intrinsic motivation, 22% (n=42; the extrinsic group) chose reasons pertaining to extrinsic motivation, and 4% (n = 8) chose other reasons for applying to medical school. The intrinsic group outperformed the extrinsic group in their GPAs, although their prior academic achievements did not differ significantly. The intrinsic group showed significantly higher levels of academic interest and also performed better in the admission interviews.

**Conclusions:**

Our study illustrates differences in medical students’ academic interest and performance across career choice motivations. Further research is warranted to establish the predictive power of medical students’ career choice motivation and academic interest on their academic performance.

## Introduction

The learner’s motivation is a critical component of the learning process and research indicates that it influences his or her learning and performance.^1^^,^^2^ Motivation is also known to be a predictor for students’ persistence or continuation in a study and psychological well-being, such as distress and burnout.^2^^,^^3^ Accordingly, research in medical education has shown that medical students’ motivation is associated with their academic achievement.^4^^-^^6^

Among several constructs on motivation is the student’s motivation for pursuing a medical career when entering medical school, which is referred to as career choice motivation.^3^ Career choice motivation is one of the attributes that are often looked into in selecting medical students. As healthcare students tend to bring more unique intentions and motivations for learning than is commonly seen in other university students,^7^it can be argued that it is important to understand the impact of career choice motivation on their learning and performance. Although research has shown that career choice motivation is related to burnout in medical students^3^ and suggests a link between their career choice motivations and approaches to learning,^8^ empirical evidence is lacking on the relationship between medical students’ career choice motivation and their academic performance.

The literature suggests career choice motivation involves intrinsic and extrinsic motivations.^1^ Those intrinsically motivated pursue an activity for personal interest or enjoyment, and those who are extrinsically motivated engage in an activity for a desirable outcome – i.e., to obtain a reward - or because of pressure from others.^9^ In addition, interest is closely linked to an individual’s intrinsic motivation.^9^^,^^10^ Accordingly, medical students’ academic interest is instrumental to understanding their motivation. However, research has not determined whether medical students’ career choice motivations are associated with their academic interest. This study therefore aimed to investigate differences in medical students’ academic interest and performance across career choice motivations.

## Methods

### Participants and settings

We conducted a cross-sectional study in a sample (n = 207) of medical students at Dongguk University medical school (DUMS), a private medical school in Korea, stratified by year of medical course. All of the students in the basic medical program at DUMS were invited to participate in the study. About one-third of the students were undergraduate-entry, and two-thirds were graduate-entry students. Students of both entry-levels were in the same four-year medical curriculum, in which undergraduate-entry students take a two-year premedical program that precedes the medical program.

Institutional review board (IRB) approval was not requested for the present study, because it was part of the annual survey of students that pertain to their learning outcomes, which fell under the general exemption from our IRB for educational outcomes data. Participation was voluntary and consent was implied with the return of the survey as responses were collected anonymously.

### Instrument and procedures

A self-report questionnaire was administered in April, 2014. The questionnaire consisted of 7 items on participant demographics, one item on the respondent’s levels of academic interest, and one item on career choice motivation. The item on career choice motivation was adapted from the instrument, “Career Choice Motivation” by Pagnin et al.^3^ In this item, respondents are asked to choose one main reason for applying for medical school among seven possible response options. These response options consist of two components of motivation for applying to medical school: intrinsic motivation with three options (interest in human relationships, intellectual curiosity, and altruism) and extrinsic motivation with four options (economic concern, professional profile, illness or death experiences, and influence of someone). The original English version had previously been translated into Korean by one of the authors, reviewed by experts and pilot tested in previous years for validation.

Some response options in the item on career choice motivation were modified from the original version for adaptation to the Korean context. Two response options in the extrinsic motivation component of the original version, “good chance of financial gain”, and “self-employed professional and social prestige”, were merged into one category and named “social prestige and good chance of financial gain”. A new response option, “obtained a very good score in the college entrance exam”, was added into the extrinsic motivation component as it was a frequently selected answer among reasons for entering medical school in previous surveys. An “others” option was also added to solicit responses that were not covered by the given response options, which gives a total of 8 response options.

The item on academic interest was adapted from the instrument developed by Kim et al.^11^ This item comprises the question, “How would you rate your level of interest in the study of medicine?” with responses given on a five-point Likert scale ranging from 1 = “very uninterested”, to 5 = “very interested”.

Independent t-test was performed to compare the student’s level of academic interest, academic performance, and admission scores between the two groups. Additionally, distributions of participant backgrounds (i.e., gender, entry-level, undergraduate majors) were compared across groups of different career choice motivations using chi-square test. IBM-SPSS version 20 was used and the significance level was 0.05 for the statistical analysis.

## Results

### Participants’ demographics and their career choice motivations

A total of 195 students completed the questionnaire (94% response rate) comprising of 78 (40%) female and 117 (60%) male; 76 (39%) undergraduate-entry and 119 (61%) graduate-entry. Participant ages ranged from 19 to 37 years (M=25.4, SD = 3.58).

For the main reason for applying to medical school, 74% (n=145; the intrinsic group) of the participants chose those related to intrinsic motivation, 22% (n=42; the extrinsic group) chose those pertaining to extrinsic motivation, and 4% (n=8) chose other reasons. The most frequently selected answer was “intellectual curiosity (n=76, 39%)”, followed by “personal illness / illness or death of family members (n =36, 18%)”, and “social prestige and good chance of financial gain (n=27, 14%)”.

There were no differences in the distributions of participants’ career choice motivation across gender (χ^2^ = 0.99, p = 0.61) or across entry-levels (χ^2^=15.30, p=0.23). Participant ages did not differ between the intrinsic and extrinsic groups (M=26.7 vs. 26.8, *t* =0.07, p=0.94).

### Comparisons of participants’ academic interest and performance

Table 1 compares the participants’ Grade Point Averages (GPAs) across groups of different career choice motivations by year of study. The intrinsic group outperformed the extrinsic group in all years in the medical program, as measured by mean GPA. This difference was significant in Years 2 and 3, although the effect sizes were moderate to small (d = 0.48 and 0.12, respectively). Additionally, the two groups differed in their levels of interest in the study of medicine (*t* = 2.94, p < 0.01) with a moderate effect size (d = 0.51).

Participants’ admission scores were compared between the two groups to investigate differences in their baseline performance. Admission scores were obtained for graduate-entry students (n=119) only, because such data were not available for undergraduate-entry students. The two groups did not differ in their undergraduate GPAs and Korean medical school entrance exam (Medical Education Eligibility Test: MEET) scores, but interview scores were significantly higher in the intrinsic group (*t*=3.00, p < 0.01).

**Table 1:** Comparison of participants’ academic interest, GPAs, and admission scores across different career choice motivations (n = 195)

Participants	The intrinsic group (Mean± SD)	The extrinsic group (Mean ± SD)	*t *(*p*)
Interest in the study of medicine	4.03 ± 0.83	3.60 ± 0.84	2.94 (< 0.01)
Medical School GPAs			
Year 1	3.13 ± 0.54	3.05 ± 0.51	0.56 (0.57)
Year 2	3.27 ± 0.52	3.03 ± 0.49	2.03 (0.04)
Year 3	3.51 ± 0.38	3.31 ± 0.31	1.93 (0.05)
Year 4	3.64 ± 0.30	3.46 ± 0.25	1.86 (0.65)
Admission scores^*^			
Undergraduate GPAs	91.56 ± 3.30	91.29 ± 2.91	0.96 (0.34)
Medical School Entrance Exam (MEET) Scores	167.32 ± 20.08	171.82 ± 24.02	0.51 (0.61)
Interview scores	100.76 ± 1.08	93.77 11.60	3.00 (< 0.01)

*Admission scores are for graduate-entry students only (n = 119)

## Discussion

Our study investigated the relationship between medical students’ career choice motivation and their academic performance. With the distributions of individual backgrounds (i.e., gender, entry-levels, and ages) and prior academic achievements not being different between students with different career choice motivations, our findings indicate that career choice motivation in medical students may account for differences in their academic performance.

Approximately two-thirds of the students in our study reported they were intrinsically motivated in applying for medical school. This finding is similar to that of the study of Brazilian students by Pagnin et al.^3^ Still, there were differences in the distributions of student responses across these two studies. In our study, the most frequently selected answer to the reason for applying to medical school was intellectual curiosity, whereas in Pagnin’s,^3^ it was professional profile. These findings suggest that there are differences in medical students’ career choice motivations according to the cultural or social contexts that they are in.

Moreover, we found differences in students’ perceived levels of academic interest between the groups of different career choice motivations. Motivational theories suggest that intrinsic motivation is positively associated with academic performance.^12^ Therefore, it can be interpreted that those who are intrinsically motivated in their career choice in medicine likely have higher intrinsic interest in learning medicine than those who are extrinsically motivated, and that higher intrinsic interest in learning may be linked to higher academic achievement. Still, our study did not explore reasons for the differences in the students’ levels of academic interest across different career choice motivations as it was beyond the scope of this study. Future research is warranted to investigate relationships between medical students’ career choice motivation and their academic interest.

Our study had several limitations. First, this was a preliminary study into career choice motivation using a relatively small sample from one institution. Therefore, future research with a larger sample from multiple institutions is warranted to enhance the generalizability of this study. Second, our study sample is relatively unique in that two different tracks of students – i.e., undergraduates and graduates - are in the same cohorts. Although our study revealed no differences in baseline performance between graduates and undergraduates, past studies suggests there are differences between these two groups in their motivational profiles and learning outcomes.^13^ Therefore, readers need to take this into account in generalizing our findings into their own contexts. Third, although this study found a relationship between medical students’ career choice motivation, their academic interest and performance, it does not reveal the extent to which career choice motivation and academic interest account for the differences in their academic performance. Further multivariate analysis is recommended to establish the predictive power of the medical students’ career choice motivation and academic interest on their academic performance.

## Conclusions

Our study illustrates differences in medical students’ academic interest and performance across career choice motivations. Our findings suggest that understanding medical students’ career choice motivation may enhance our knowledge of factors that influence medical students’ learning and performance. Such an understanding can shed us light on how to select students who are likely to succeed in medical school and how to better facilitate their learning.

### Conflict of Interest

The authors declare that they have no conflict of interest.
